# The Effect of Veteran Race and Socioeconomic Status on Enrollment in Remote Patient Monitoring for Hypertension: Retrospective Observational Cross-Sectional Study

**DOI:** 10.2196/78423

**Published:** 2026-03-09

**Authors:** Hannah Robin Friedman, Hillary J Mull, Jenice Ria Guzman-Clark, Daniel J Sturgeon, Marva V Foster

**Affiliations:** 1 Center for Health Optimization and Implementation Research VA Boston Healthcare System Boston, MA United States; 2 Chobanian and Avedisian School of Medicine Boston University Boston, MA United States; 3 Southern Arizona VA Health Care System Tucson, AZ United States; 4 Stanford Medicine Palo Alto, CA United States; 5 Health Economics Resource Center VA Palo Alto Health Care System Palo Alto, CA United States

**Keywords:** hypertension, veterans’ health, remote patient monitoring, telehealth, health equity

## Abstract

**Background:**

Black veterans and veterans from lower socioeconomic backgrounds are more likely to have uncontrolled hypertension. One potential explanatory factor is reduced access to specific treatments that result in improved chronic disease management. In the Veterans Health Administration (VHA), veterans with hypertension may enroll in a remote patient monitoring (RPM) program, which consists of patient education, daily home blood pressure (BP) monitoring, health coaching, and case management. Barriers for socioeconomically disadvantaged patients may exist for similar programs in other health systems; however, the VHA is an integrated health care system, and these barriers may differ for veteran populations.

**Objective:**

The objective of this study was to assess the relationship between veteran race and neighborhood socioeconomic status and the likelihood of enrolling in the VHA RPM program.

**Methods:**

The study sample included VHA-enrolled veterans with a diagnosis of hypertension (average BP >130/80 mm Hg on ≥2 BP readings) between fiscal years 2020 and 2023. We ran random-effects logistic regression models to assess the relationship between veteran race and Area Deprivation Index and RPM enrollment each year, controlling for potential demographic and clinical confounders. For sensitivity analysis, we limited our sample to veterans with stage 2 hypertension (BP >140/90 mm Hg) and on antihypertensive medication.

**Results:**

Overall use of RPM was low, with only 4.1% (56,553/1,390,995; 95% CI 4%-4.1%) of veterans being enrolled in RPM. Black veterans, who represented 26.6% (n=35,096) of all veterans, were more likely (odds ratio [OR] 1.65, 95% CI 1.59-1.70) to enroll in RPM compared to White veterans. Asian American or Pacific Islander veterans were less likely to enroll (OR 0.83, 95% CI 0.74-0.94). We found no meaningful association between Area Deprivation Index and RPM enrollment (OR 1.00, 95% CI 0.99-1.00). When limiting our sample to those with stage or grade 2 hypertension, we found a similar association (OR 1.61, 95% CI 1.50-1.72) between Black race and RPM enrollment but no significant association with Asian or Pacific Islander race (OR 1.02, 95% CI 0.80-1.29).

**Conclusions:**

Prior research on RPM in veterans has examined duration or outcomes of RPM enrollment but not the probability of initial enrollment. We found higher enrollment rates in the VHA RPM program among Black veterans but slightly lower enrollment among Asian American or Pacific Islander veterans. Higher enrollment among Black veterans and among those with higher comorbidity burden suggests that the VHA RPM program is successfully reaching those who could most benefit, despite low overall enrollment. Given the low enrollment in RPM, future research should focus on improving uptake among veterans who could additionally benefit from the program. Non-VHA systems, particularly those serving low-income or socioeconomically disadvantaged areas, should explore subsidized or free RPM programs for eligible patients similar to the VHA’s no-cost model for veterans.

## Introduction

Remote patient monitoring (RPM) is a form of telehealth that allows clinicians to track patient vital signs and health metrics outside traditional health care settings [1]. This can include using wireless-enabled blood pressure (BP) cuffs, thermometers, pulse oximeters, scales, and glucometers to transmit vital signs to a nurse from the patient’s home on a regular basis. Integrating RPM into chronic disease management can potentially improve patient quality of life and mitigate health care costs by enabling the timely detection of health deterioration, thereby reducing unnecessary emergency department visits and hospital admissions [2,3]. The Veterans Health Administration (VHA) is an integrated health care system that provides health care for more than 9 million of the 18 million veterans in the United States [4]. As a comprehensive health care system, the VHA offers RPM services at no cost to veterans and has existed since the early 2000s serving veterans with a variety of chronic conditions [5]. The VHA RPM model is unique due to its nationwide coverage, centralized funding, and focus on a specific patient population. The essence of RPM, as implemented in the VHA, is ongoing assessment, monitoring, patient education, health coaching, and nurse case management of veterans in their place of residence. It also includes the provision of appropriate information to the veterans’ primary care team or specialty care team, as well as the health care system, to enable timely care [5].

Veterans targeted for RPM are those who have high health service use (eg, frequent emergency room visits) due to chronic diseases, who have uncontrolled chronic diseases, and who agree to participate in daily submission of health data for monitoring [6,7]. Referrals can be made by any VHA-licensed independent clinician (physician, nurse practitioner, or physician assistant) or other clinicians when following an approved local protocol. Once enrolled, veterans are expected to submit vital sign and other relevant biometric data every day and respond to daily disease-specific questions.

Hypertension, or high BP, is one of the most common chronic medical conditions managed by the VHA RPM program [8]. Hypertension is a major contributor to cardiovascular morbidity and mortality in the United States and remains one of the most important modifiable cardiovascular risk factors [9]. Prevalence data indicate that 47% of adults in the United States have hypertension, which is equivalent to an estimated 122 million adults [10]. More than half of these individuals with hypertension have BP that is inadequately controlled [11]. There are racial and ethnic differences in hypertension prevalence and treatment in the United States, with more Black, Hispanic, and Asian adults having poor control of their BP compared to White adults despite Black adults having a higher prevalence of hypertension [12]. Hypertension is the most common chronic condition among patients in the VHA. Recent research has found that 87% of veterans have high BP and, of those, most (66%) are considered to have uncontrolled hypertension [13]. Effective control of high BP is of key importance for reducing the risk of hypertension-related cardiovascular disease complications.

However, several factors have been found to impact hypertension control, key among them being patient residence in socioeconomically disadvantaged neighborhoods [14-16]. In the VHA, Black veterans have the poorest control rates for hypertension compared to other ethnic backgrounds even after adjusting for access to care and socioeconomic status [17]. Black veterans also have considerably higher rates of cardiovascular-related hospitalizations, an indicator of poor hypertension control [18]. RPM has been posited as a potential intervention to decrease this racial and ethnic disparity in hypertension control and has been shown to improve hypertension control in patients of a large health care organization [3]; however, a small pilot study that compared a sample of primary care patients who mostly comprised minority groups or groups with low socioeconomic status who used RPM to a control group who did not use RPM found no difference in BP control after 90 days, but found that RPM enrollment was associated with decrease in all-cause clinic visits [19].

There is no singular socioeconomic determinant implicated in disadvantaged neighborhoods. Multidimensional factors such as economic (eg, low income and blue-collar jobs), social (eg, low educational level, nonvoter status, and high unemployment), and physical (eg, graffiti and low-rent housing) conditions, among others, can influence a neighborhood [20,21]. These neighborhood-level characteristics have been increasingly recognized as playing a critical role in health [22]. The use of RPM has been suggested as a potential solution to overcome the geographical limitations of health care services [23,24]. However, a review of existing literature on non-VHA populations has shown that some studies indicate the expected directionality (ie, greater neighborhood socioeconomic disadvantage is associated with decreased use of RPM), whereas others report mixed and null findings [25-28]. The pathway between neighborhood deprivation and RPM use for hypertension management in the VHA is poorly understood. Previous VHA studies have examined adherence to RPM and enrollment rates in RPM but have not examined the role of neighborhood deprivation on RPM enrollment [8,29-31]. Non-VHA studies have identified barriers to RPM among Black patients, including lower overall participation in clinical trials and reduced precision and accuracy of wearable devices on people with darker skin tones [32,33]. The overall evidence points to the fact that living in a socioeconomically disadvantaged neighborhood may impact hypertension and possibly RPM use in non-VHA populations.

What is not known is whether neighborhood deprivation impacts enrollment in RPM among veterans; such knowledge will identify populations for whom appropriate interventions can be implemented. Therefore, the purpose of this study was to investigate whether veteran race or residence in a socioeconomically disadvantaged neighborhood was associated with the probability that a patient with hypertension will enroll in the VHA RPM program.

## Methods

We used the Strengthening the Reporting of Observational Studies in Epidemiology (STROBE) reporting guidelines to ensure completeness and accuracy when reporting our methods and results [34].

### Population and Sample

This retrospective cross-sectional study included veterans with a diagnosis of hypertension between October 1, 2019, and September 30, 2023. We used data from the VHA’s Corporate Data Warehouse to identify all covariates and outcomes for this study. Our initial sample consisted of all veterans with an *International Classification of Diseases, 10th Revision*, diagnosis code for hypertension on or before October 1, 2022 [35]. We excluded observations in which the veteran was not enrolled in the VHA at the start of the fiscal year (FY), did not have a diagnosis of hypertension before the start of the FY, or died before the end of the FY. We then limited our sample to veterans with uncontrolled hypertension.

### Definition of Uncontrolled Hypertension

The American College of Cardiology and American Heart Association have defined hypertension as a BP of at least 130/80 mm Hg over a minimum of 2 BP readings [36,37]. To ensure that our sample consisted of those with hypertension in accordance with clinical practice guidelines, we limited the study sample to veterans with ≥2 days of recorded BP readings in the prior year and with an average BP reading of at least 130/80 mm Hg across all readings. For sensitivity analysis, we further limited the sample to those with an average BP of at least 140/90 mm Hg (hypertension stage or grade 2) despite being prescribed antihypertensive medications [37].

### Measures

#### Outcome

Our outcome was any enrollment in the VHA’s hypertension RPM program each year. Veterans were considered to have enrolled in RPM if any encounter had an RPM VHA stop code (683, 684, 685, or 686) included as a primary or secondary stop code in electronic health record data. VHA stop codes are administrative codes used to categorize and track outpatient clinical services and have been used in previous studies [38,39]. We did not differentiate between new enrollment or continued use of RPM from patients enrolled in RPM during the prior year. We also did not consider the duration of enrollment as the focus of this study was veteran enrollment in the RPM program.

#### Primary Independent Variables

For enrollment-adjusted regression models, our primary independent variables comprised veteran race (White; Black; Asian American, Native Hawaiian, Pacific Islander; American Indian, Alaska Native; and other race, unknown, or multiracial) and the Area Deprivation Index (ADI) of the census block area of the veteran’s primary address. When possible, veteran race in the Corporate Data Warehouse is self-reported, and prior research has found high agreement between Corporate Data Warehouse race and self-reported race and ethnicity [40]. The ADI is an established composite measure of an area’s overall socioeconomic vulnerability and includes measures of median home value, poverty rate, and education, among others. For analysis, the unit of change was a 10-percentile change in ADI national ranking.

#### Control Variables

We controlled for patients’ overall health, specific comorbidities, age, sex, rurality, VHA priority group, and distance to the nearest VHA facility. Veterans’ overall health was assessed using the Elixhauser Comorbidity Index, and we controlled separately for a diagnosis of diabetes mellitus due to complications associated with comorbid diabetes and hypertension, as well as the presence of a separate RPM program for veterans with diabetes. We also controlled for the type of hypertension (uncomplicated or with complications) as defined by the Elixhauser Comorbidity Index [41]. We described veterans’ rurality using the VHA classification of urban, rural, and highly rural, which is originally based on Rural-Urban Commuting Area codes. A VHA priority group is a category assigned to veterans to determine the amount, if any, they may have to pay for health care. Veterans are classified into priority groups 1 to 8. We aggregated priority groups 1 to 3 (service-connected disability), priority group 4 (non–service-connected catastrophic disability or receiving VHA aid benefits), priority group 5 (low income or Medicaid eligible), and priority groups 6 to 8 (service history and copayments required for some services). All variables reflected the status of the patient at the beginning of the FY. For covariates for which data were missing (rurality, priority group, ADI, and comorbidities), we used multiple imputation to generate replacement values. Data processing and analysis were conducted using SAS Enterprise Guide (SAS Institute) and Stata (version 18; StataCorp).

### Analysis

For descriptive analysis, we assessed the veteran characteristics by RPM enrollment; we used chi-square tests (for categorical variables) and Wilcoxon rank sum tests (for continuous variables) to assess the significance of differences between White veterans, Black veterans, and veterans of other races. We ran random-effects logistic regression models to assess the likelihood of a veteran being enrolled in RPM each year from FYs 2020-2023, with random intercepts for each individual veteran. We separated the cohort into person-years with 1 observation per patient per year for a maximum of 4 observations per patient, although not all patients were eligible for inclusion in all 4 years. Separating patients into person-years allowed for more accurate assessments of patients’ potential confounders, such as comorbidities, age, location, and BP control, all of which could change over the course of 4 years. Using person-year as the unit of analysis also allowed us to account for the COVID-19 pandemic, which began in FY 2020 and substantially impacted health care use patterns, including RPM participation. We used multiple tests and metrics to assess model goodness of fit using log-likelihood ratio tests and also tested for potential collinearity using variance inflation factors.

### Sensitivity Analysis

For sensitivity analysis, we limited our sample to those with stage or grade 2 hypertension (average BP reading>140/90 mm Hg) and presence of at least one hypertension medication in the 6 months before the start of the FY to focus on those who might be most in need of closer monitoring.

### Ethical Considerations

This study was approved by the US Department of Veterans Affairs (VA) Boston Institutional Review Board (1765472-5; approval date: January 18, 2024). As this study involved only retrospective analysis of a large observational dataset derived from electronic health record data where risk was deemed minimal to study participants, we received a waiver for protected health information and a waiver of informed consent from the institutional review board. All identifiable study data were stored and analyzed on a secure server operated by the VHA Informatics and Computing Infrastructure, and no identifiable data were ever moved from that server. No identification of individual participants is included in this paper. As this study consisted of analysis of a secondary dataset, no participants received compensation.

## Results

### Overview

**Figure 1:**
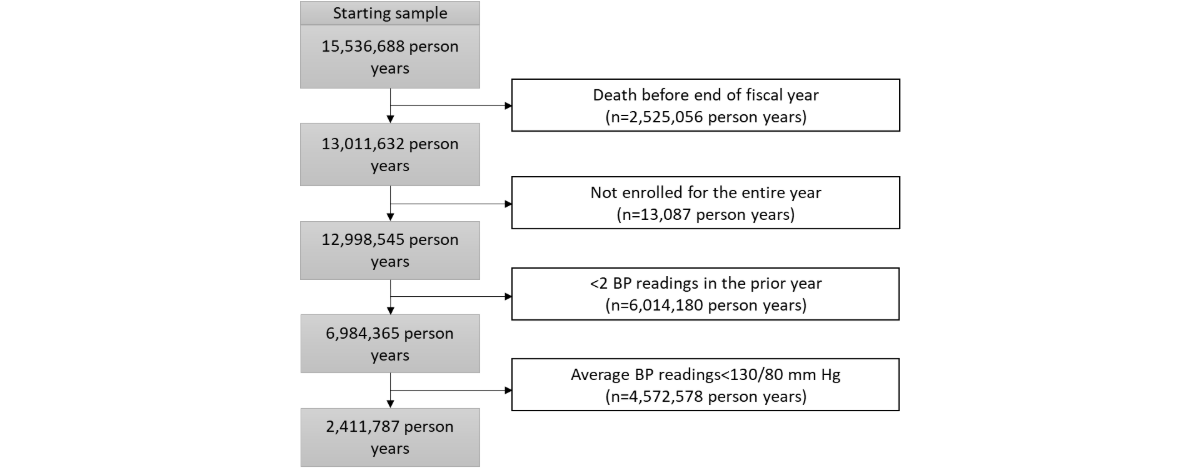
Veterans with hypertension eligible for inclusion in study sample between fiscal years 2019 and 2023.

Among those with complete covariate data and who met the eligibility criteria (Figure 1), we identified 2,411,787 total person-years during FYs 2019-2023 among 1,390,995 unique veterans.

In Table 1, we share the characteristics of all veterans in our sample stratified by whether they were ever enrolled in RPM during our study period. If veterans appeared more than once in our sample, time-varying characteristics (eg, age and comorbidity) reflect their status during the first year they were eligible for inclusion in our sample. For priority group (n=6482 person-years), rurality (n=2320 person-years), ADI (n=36,351 person-years), and comorbidity (Elixhauser Comorbidity Index; diabetes and hypertension with complications; n=58,249 person-years), we used multiple imputation to replace missing values. Overall rates of RPM use were low, with 4.1% (56,553/1,390,995; 95% CI 4.0%-4.1%) of the total sample ever enrolling in RPM. There were statistically significant differences between those who enrolled and those who never enrolled across all veteran characteristics. Those with any RPM enrollment were more likely to be Black compared to individuals with no enrollment (19,457/56,533 veterans, 34.4%, 95% CI 34.0%-34.8% vs 350,391/1,334,462, 26.3%, 95% CI 26.2%-26.3%), and there was a very small but statistically significant difference by ADI percentile (the mean percentile was 0.3 higher for those with no RPM enrollment). Veterans who ever enrolled in RPM were more likely than those who never enrolled to be in VHA priority groups 1 to 3 (service-connected disability; 39,807/56,533, 70.4%, 95% CI 70%-70.8% vs 906,648/1,334,462, 67.9%, 95% CI 67.9%-68%), reside in urban areas (38,555/56,533, 68.2%, 95% CI 67.8%-68.6% vs 864,851/1,334,462 64.8%, 95% CI 64.7%-64.9%), and have 4 or more comorbidities (22,841/56,533, 40.4%, 95% CI 40.4%-40.8% vs 369,619/1,334,462, 27.7%, 95% CI 27.6%-27.8%).

**Table 1:** Characteristics of veterans with hypertension in the study sample between fiscal years 2020 and 2023 by enrollment in remote patient monitoring (N=1,390,995).

Characteristic			Never enrolled (n=1,334,462)	Ever enrolled (n=56,533)		Total		*P* value
Area Deprivation Index, mean (SD)		58.9 (25.2)		59.2 (25.2)		58.9 (25.2)		.001
**Race n (%; 95% CI)**								<.001
	Hispanic, any race	83,061 (6.2; 6.2-6.3)		3421 (6.1; 5.9-6.3)	86,482 (6.2; 6.2-6.3)			
	Non-Hispanic American Indian or Alaska Native	8,697 (0.7; 0.6-0.7)		309 (0.5; 0.5-0.6)	9006 (0.6; 0.6-0.7)			
	Non-Hispanic Asian American and Pacific Islander	22,678 (1.7; 1.7-1.7)		747 (1.3; 1.2-1.4)	23,425 (1.7; 1.7-1.7)			
	Non-Hispanic Black	350,391 (26.3; 26.2-26.3)		19,457 (34.4; 34.0-34.8)	369,848 (26.6; 26.5-26.7)			
	Non-Hispanic White	805,018 (60.3; 60.2-60.4)		30,052 (53.2; 52.7-53.6)	835,070 (60; 60.0-60.1)			
	Non-Hispanic other	64,617 (4.8; 4.8-4.9)		2548 (4.5; 4.3-4.7)	67,165 (4.8; 4.8-4.9)			
**Fiscal year, n (%; 95% CI)**								<.001
	2020	636,001 (47.7; 47.6-47.7)		30,390 (53.8; 53.3-54.2)	666,391 (47.9; 47.8-48.0)			
	2021	229,647 (17.2; 17.1-17.3)		10,252 (18.1; 17.8-18.5)	239,899 (17.2; 17.2-17.3)			
	2022	233,225 (17.5; 17.4-17.5)		8772 (15.5; 15.2-15.8)	241,997 (17.4; 17.3-17.5)			
	2023	235,589 (17.7; 17.6-17.7)		7120 (12.6; 12.3-12.9)	242,709 (17.4; 17.4-17.5)			
**Sex, n (%; 95% CI)**								<.001
	Female	99,072 (7.4; 7.4-7.5)		5382 (9.5; 9.3-9.8)	104,454 (7.5; 7.5-7.6)			
	Male	1,235,388 (92.6; 92.5-92.6)		51,151 (90.5; 90.2-90.7)	1,286,539 (92.5; 92.4-92.5)			
**Age (y), n (%; 95% CI)**								<.001
	<65	673,661 (50.5; 50.4-50.6)		28,522 (50.5; 50.0-50.9)	702,183 (50.5; 50.4-50.6)			
	65-74	499,645 (37.5; 37.4-37.5)		21,557 (38.1; 37.7-38.5)	521,202 (37.0; 37.4-37.6)			
	75-84	124,783 (9.0; 9.3-9.4)		5172 (9.1; 8.9-9.4)	129,955 (9.3; 9.3-9.4)			
	≥85	36,371 (2.8; 2.7-2.8)		1282 (2.3; 2.1-2.4)	37,653 (2.7; 2.7-2.7)			
**Priority group, n (%; 95% CI)**								<.001
	1-3	906,648 (67.9; 67.9-68.0)		39,807 (70.4; 70.0-70.8)	946,455 (68.0; 68.0-68.1)			
	4	15,105 (1.1; 1.1-1.2)		782 (1.4; 1.3-1.5)	15,887 (1.1; 1.1-1.2)			
	5	211,435 (15.8; 15.8-15.9)		9138 (16.2; 15.9-16.5)	220,410 (15.8; 15.8-15.9)			
	6-8	201,435 (15.1; 15.0-15.2)		6806 (12; 11.8-12.3)	208,241 (15.0; 14.9-15.0)			
**Rurality, n (%; 95% CI)**								<.001
	Urban	864,851 (64.8; 64.7-64.9)		38,555 (68.2; 67.8-68.6)	903,406 (64.9; 64.9-65.0)			
	Rural	415,852 (31.2; 31.1-31.2)		15,965 (28.2; 27.9-28.6)	431,817 (31.0; 31.0-31.1)			
	Highly rural	53,759 (4.0; 4.0-4.1)		2014 (3.6; 3.4-3.7)	55,773 (4.0; 4.0-4.0)			
**Elixhauser comorbidities, n (%; 95% CI)**								<.001
	1	262,970 (19.7; 19.6-19.8)		7055 (12.5; 12.2-12.8)	270,025 (19.4; 19.3-19.5)			
	2	384,950 (28.8; 28.8-28.9)		13,145 (23.3; 22.9-23.6)	398,095 (28.6; 28.5-28.7)			
	3	316,923 (23.7; 23.7-23.8)		13,492 (23.9; 23.5-24.2)	330,415 (23.8; 23.7-23.8)			
	≥4	369,619 (27.7; 27.6-27.8)		22,841 (40.4; 40.0-40.8)	392,460 (28.2; 28.1-28.3)			
**Hypertension type, n (%; 95% CI)**								<.001
	Uncomplicated	1,224,737 (91.8; 91.7-91.8)		49,383 (87.4; 87.1-87.6)	1,274,120 (91.6; 91.6-91.6)			
	Complications^a^	109,725 (8.2; 8.2-8.3)		7150 (12.6; 12.4-12.9)	116,875 (8.4; 8.4-8.4)			
**Diabetes type 1 or 2, n (%; 95% CI)**								<.001
	No	899,914 (67.4; 67.4-67.5)		31,719 (56.1; 55.7-56.5)	931,633 (67.0; 66.9-67.1)			
	Yes	434,548 (32.6; 32.5-32.6)		24,814 (43.9; 43.5-44.3)	459,362 (33.0; 32.9-33.1)			

^a^With hypertension-related heart disease or kidney disease.

In Table 2, we share the results of our first regression model. Compared to White veterans, we found that Black veterans (odds ratio [OR] 1.65, 95% CI 1.59-1.70) and veterans of “other” race (OR 1.11, 95% CI 1.03-1.19) had higher odds of RPM enrollment. Asian American and Pacific Islander (AAPI) veterans were slightly less likely (OR 0.83, 95% CI 0.74-0.94) to enroll. We found that, while technically statistically significant, there was no meaningful association between a 10-percentile change in ADI ranking and the odds (OR 1.00, 95% CI 0.99-1.00) of RPM enrollment. We tested different variable specifications of ADI (ADI quartiles and specific cutoff points [ie, 90th percentile]) and found that those in the highest quartile of ADI (most vulnerable) had slightly lower odds of RPM enrollment compared to those in the first quartile (least vulnerable) but that the magnitude (OR 0.93, 95% CI 0.90-0.97) was still quite small (Multimedia Appendix 1).

**Table 2:** Odds ratios from logistic regression modeling the odds of remote patient monitoring enrollment among veterans with hypertension from fiscal years 2020 to 2023.

Characteristic			Odds ratio (95% CI)
**Race**			
	Hispanic, any race	1.09 (1.02-1.16)	
	Non-Hispanic American Indian or Alaska Native	0.90 (0.74-1.09)	
	Non-Hispanic Asian American and Pacific Islander	0.83 (0.74-0.94)	
	Non-Hispanic Black	1.65 (1.59-1.70)	
	Non-Hispanic White	—^a^	
	Non-Hispanic other	1.11 (1.03-1.19)	
Area Deprivation Index (per 10-percentile change)			1.00 (0.99-1.00)
**Fiscal year**			
	2020	—	
	2021	1.22 (1.18-1.26)	
	2022	1.12 (1.08-1.16)	
	2023	1.30 (1.26-1.34)	
**Sex**			
	Female	—	
	Male	0.72 (0.69-0.76)	
**Age (y)**			
	<65	—	
	65-74	1.04 (1.01-1.08)	
	75-84	1.12 (1.06-1.17)	
	≥85	0.95 (0.87-1.05)	
**Priority group**			
	1-3	—	
	4	1.20 (1.05-1.36)	
	5	1.01 (0.97-1.05)	
	6-8	0.83 (0.79-0.87)	
**Rurality**			
	Urban	—	
	Rural	0.91 (0.88-0.94)	
	Highly rural	0.94 (0.87-1.02)	
**Elixhauser comorbidities**			
	1	—	
	2	1.24 (1.18-1.29)	
	3	1.63 (1.56-1.71)	
	≥4	2.47 (2.36-2.58)	
**Hypertension type**			
	Uncomplicated	—	
	With complications^b^	1.57 (1.51-1.64)	
**Comorbid diabetes**			
	No	—	
	Yes	1.47 (1.42-1.51)	

^a^Reference group.

^b^With hypertension-related heart disease or kidney disease.

In addition to veteran race, we found that male veterans were less likely (OR 0.72, 95% CI 0.69-0.76) to enroll in RPM and those between the ages of 65 and 84 years were slightly more likely to enroll in RPM than younger veterans. Among clinical covariates, veterans with diabetes (OR 1.47, 95% CI 1.42-1.51), hypertension-associated complications (eg, heart disease or kidney disease; OR 1.57, 95% CI 1.51-1.64), and more chronic health conditions (OR 1.24-2.47) were all significantly associated with higher odds of RPM enrollment.

### Sensitivity Analysis

For sensitivity analyses, we further limited our sample to those with grade or stage 2 hypertension (average BP>140/80 mm Hg across ≥2 BP measurements) and who also were prescribed at least one antihypertensive medication. These requirements further limited our sample to 464,971 person-years across 348,265 unique veterans, or 19.3% (464,971 /2,411,782 person-years) of the sample in the original analysis. Among these veterans, compared to the initial sample, a higher proportion (17,762/348,265, 5.1%, 95% CI 5.0%-5.2%) were enrolled in RPM.

In the adjusted analysis for this population (Table 3), Black veterans still had higher odds (OR 1.61, 95% CI 1.50-1.72) of being enrolled in RPM compared to White veterans. There was no longer a statistically significant association between AAPI race and RPM enrollment. However, there was a slightly larger association between “other” race and RPM enrollment (OR 1.19, 95% CI 1.03-1.37). For ADI, the association with RPM enrollment remained very small. While most variables that were significant in the first model remained so in the second model, the magnitude was attenuated; for example, those with 4 or more comorbidities now had only 2.04 (95% CI 1.86-2.24) times the odds of RPM enrollment compared to 2.47 (95% CI 2.36-2.58) times the odds among the larger sample. The magnitude of other covariates also shifted, with some associations now not significant either due to differences in the patient population or due to the smaller sample size.

**Table 3:** Odds ratios from logistic regression modeling the odds of remote patient monitoring enrollment among veterans with stage 2 hypertension (blood pressure >140/90 mm Hg) and antihypertensive medication use from fiscal years 2020 to 2023.

Characteristic		Odds ratio (95% CI)
**Race**		
	Hispanic, any race	1.27 (1.12-1.45)
	Non-Hispanic American Indian or Alaska Native	1.12 (0.77-1.65)
	Non-Hispanic Asian American and Pacific Islander	1.02 (0.80-1.29)
	Non-Hispanic Black	1.61 (1.50-1.72)
	Non-Hispanic White	—^a^
	Non-Hispanic other	1.19 (1.03-1.37)
Area Deprivation Index (per 10-percentile change)		0.99 (0.98-1.00)
**Fiscal year**		
	2020	—
	2021	1.14 (1.05-1.22)
	2022	1.07 (0.99-1.15)
	2023	1.26 (1.17-1.36)
**Sex**		
	Female	—
	Male	0.68 (0.61-0.75)
**Age (y)**		
	<65	—
	65-74	0.98 (0.92-1.05)
	75-84	1.07 (0.93-1.02)
	≥85	1.03 (0.77-1.37)
**Priority group**		
	1-3	—
	4	1.25 (0.93-1.68)
	5	0.94 (0.86-1.02)
	6-8	0.92 (0.84-1.00)
**Rurality**		
	Urban	—
	Rural	0.94 (0.88-1.01)
	Highly rural	0.80 (0.67-0.95)
**Elixhauser comorbidities**		
	1	—
	2	1.18 (1.08-1.29)
	3	1.48 (1.35-1.63)
	≥4	2.04 (1.86-2.24)
**Hypertension type**		
	Uncomplicated	—
	With complications^b^	1.63 (1.51-1.77)
**Comorbid diabetes**		
	No	—
	Yes	1.36 (1.28-1.45)

^a^Reference group.

^b^With hypertension-related heart disease or kidney disease.

## Discussion

### Principal Results

RPM for hypertension may provide disease management support; however, we found that only 4.1% (56,533/1,390,995) of potentially eligible veterans were ever enrolled during our study period. This finding aligns with the relatively low enrollment in RPM for other chronic diseases within the VHA [38,42]. While having different inclusion criteria, other studies examining RPM in the VHA have found relatively low enrollment [38]. In our retrospective national cross-sectional study of veterans with hypertension from FYs 2020-2023, we found mixed data on racial disparities in RPM enrollment and no association with the socioeconomic vulnerability of the neighborhood where the veterans lived. Black veterans and veterans of other races, including those identifying as multiracial or with unknown race, were more likely to be enrolled in RPM, whereas AAPI veterans were less likely to enroll. We found no meaningful difference in the likelihood of enrollment by neighborhood socioeconomic characteristics.

### Comparison With Prior Work

Controlling for potential demographic and clinical confounders, we found that Black veterans were more likely than White veterans to enroll in RPM for hypertension and AAPI veterans were less likely to enroll. Previous research found that overall awareness of hypertension and hypertension control was lower among Asian American patients compared to non-Hispanic White patients, although there was considerable variation by ethnic group [14,43]. Prior research involving the use of RPM among patients without hypertension found that Black patients were more likely to be engaged in the program [44], whereas other research on different populations (nonveterans and those with heart failure) found that female patients and Black patients were less likely to enroll in RPM [32]. In our study, veterans with multiple comorbid conditions and those with diabetes or hypertension-associated complications were more likely to enroll in RPM. These findings indicate that VHA RPM is reaching the appropriate population as those targeted for additional support by VHA RPM are patients who have high health service use (eg, frequent emergency room visits) due to multimorbidity [5,31].

We found no meaningful difference in enrollment in RPM by ADI. This finding held true even when we tested different approaches to modeling, such as ADI quartiles or specified cutoffs. This finding supports previous research among rural and low-income patients that found that socioeconomic status was not a barrier to RPM use [45]. However, it contrasts other research that found that those in more disadvantaged neighborhoods are less likely to have access to and enroll in RPM [44,46-48]. These studies have often cited factors such as differential access to care, low health literacy, limited access to technology, or broadband availability (ie, internet access) as barriers to RPM use [49-51]. Barriers to RPM in the VHA are in part ameliorated because access to RPM is available to all eligible veterans at no cost and the technology used in the VHA does not necessitate broadband availability [5]. This may explain why ADI may not have impacted RPM use in our study population.

Among a smaller subset of patients with documented stage or grade 2 hypertension despite the use of hypertension medications, we still found that Black veterans were more likely than White veterans and AAPI veterans to enroll in RPM, but the magnitude of the association shrank, as it did for most clinical covariates. Perhaps the reason Black veterans were more likely to enroll in RPM was linked to their health and morbidity status as Black veterans are more likely to report circulatory-related chronic conditions than non-Hispanic White veterans [52]. Previous research has found that patients with hypertension and concomitant multimorbidity incur higher health care costs and have more complex health care needs [53-55]. While Black veterans were more likely than White veterans to enroll in the VHA RPM program, future research is needed to encourage uptake of RPM and determine whether RPM is equally effective across all participants. Additionally, given the low (approximately 4%) overall rate of RPM enrollment, RPM is likely one of many tools and approaches required to improve hypertension control in the larger population. Related interventions, such as video BP visits, have successfully reduced BP in veterans, but the intervention did not assess for whom the intervention was most effective [56]. Recent literature on hypertension among veterans shows that Black veterans, followed closely by American Indian and Alaska Native veterans, have the lowest overall rates of hypertension control [17]. Studies of RPM programs that specifically evaluate their effect on racial disparities in hypertension outcomes are promising but limited [57].

### Limitations

While our sample included more than 1 million unique veterans over 4 years, our findings may not be generalizable to other populations. Veterans are not representative of the general American population as they are much more likely to be male, White, and older, although younger veteran populations are more diverse. Furthermore, we limited our sample to those with ≥2 BP readings on ≥2 occasions with an average BP of >130/80 mm Hg. This approach ensured that our study cohort had an active case of hypertension according to clinical guidelines but likely nonrandomly eliminated veterans who were healthier (and had fewer medical encounters in which BP was taken) and those who were less reliant on VHA care and less likely to have BP readings documented in VHA data. This approach further limits the generalizability of our study to the veteran population as a whole. We also excluded veterans who died, which likely eliminated veterans with poorer health, which also limits the implications of our study. Our study also only evaluated the VHA’s RPM program, whereas other similar programs to monitor and control hypertension may be structured differently. Participants of other non-VHA programs may also have different barriers (such as lack of insurance coverage and changes in health care systems) that are less present in the VHA veteran population, where many, though not all, veterans receive much of their care in the same system with low or no copayments. Those in lower priority groups (6-8) were the least likely to enroll in RPM, perhaps because they received less care at the VHA overall and were subject to copayments for some services [58,59].

Additionally, while we controlled for a number of clinical and demographic characteristics, there may still be some confounding information that we were unable to capture. Although widely used with established best practices at identifying the most accurate value, Corporate Data Warehouse data on race do not always consist of veteran self-reported race. Primary care provider awareness of RPM as an option for their patients may vary substantially, and there could also be differences between facilities in terms of awareness, uptake, and support for this program for veterans. Future research will examine these other factors. This study also did not evaluate how long veterans stayed in the RPM program, only whether they enrolled. Recent research has examined enrollment duration, and data on reasons for enrollment and disenrollment are stored externally to the VHA electronic health record by a third-party server, which was not accessible for this study [8]. Finally, while there are limitations to the use of ORs for reporting regression results, for rare outcomes such as ours, ORs approximate the risk ratio and eliminate these potential concerns [60].

### Conclusions

Controlling for potential confounders, Black veterans were more likely than White veterans to be enrolled in the VHA hypertension RPM program, but we found no difference in RPM enrollment by neighborhood socioeconomic status. These results were consistent when limited to a smaller sample of veterans with hypertension that remained uncontrolled despite the use of antihypertensive medications. Prior research has assessed the duration of enrollment and outcomes after enrolling, but no prior studies have examined the probability of any enrollment in the RPM program for controlling hypertension in a veteran population. In an integrated health care system in which most patients do not pay for health care services, we found that patients from socioeconomically disadvantaged areas and Black veterans were not less likely to be enrolled in RPM. Our study results demonstrate that, within the VHA-enrolled veteran population, RPM is reaching an appropriate patient population (ie, patients with more comorbidities and patients with stage or grade 2 hypertension). However, overall enrollment was low, suggesting that RPM could still benefit additional patients who are not currently enrolled.

For non-VHA settings, reducing or eliminating financial barriers may improve access for similar nonveteran populations. As overall enrollment in RPM for hypertension was low, future research should focus on improving uptake among veterans who could additionally benefit from the program, including White and AAPI veterans. While the VHA is a unique health care system in the United States, being the largest integrated health care system in the country and serving a unique population, outside the VHA, RPM programs may be able to effectively reach minority populations and those living in socioeconomically disadvantaged areas. This study demonstrated that RPM use was not impacted by neighborhood socioeconomic status when the use of those services was either fully or partially paid for and the technology was not reliant on broadband availability. Non-VHA systems, particularly those serving low-income or socioeconomically disadvantaged areas, should explore subsidized or free RPM programs for eligible patients similar to the VHA’s no-cost model for veterans.

## Appendix

Multimedia Appendix 1 Sensitivity analysis.

## Abbreviations

AAPI: Asian American and Pacific Islander
ADI: Area Deprivation Index
BP: blood pressure
FY: fiscal year
OR: odds ratio
RPM: remote patient monitoring
STROBE: Strengthening the Reporting of Observational Studies in Epidemiology
VA: US Department of Veterans Affairs
VHA: Veterans Health Administration

## Data Availability

The datasets generated or analyzed during this study are not publicly available due to Veterans Health Administration policies but are available from the corresponding author on reasonable request. All requests are subject to compliance with all security requirements of the US Department of Veterans Affairs data sharing agreements.
