# Supplementary material for: The Effect of Veteran Race and Socioeconomic Status on Enrollment in Remote Patient Monitoring for Hypertension: Retrospective Observational Cross-Sectional Study
Source: J Med Internet Res. 2026 Mar 9;28:e78423. doi: 10.2196/78423 (PMC13010073; doi:10.2196/78423)
Supplement: Multimedia Appendix 1 [file jmir_v28i1e78423_app1.docx]

**Supplemental Material**

**Supplemental Table 1. Analysis of Using Different ADI Variable Operationalizations**

| ADI Variable Definition | OR | 95% CI |
| --- | --- | --- |
| ADI (per 10 percentile increment) | 0.99 | 0.99-1.00 |
| ADI ≥90^th^ percentile (vs. < 90^th^ percentile) | 0.97 | 0.93-1.01 |
| Study Sample ADI Quartile |  |  |
| 1^st^ quartile (ADI Range: 1-37) | -- | -- |
| 2^nd^ quartile (ADI Range 38-59) | 0.98 | 0.94-1.02 |
| 3^rd^ quartile (ADI Range 60-79) | 0.97 | 0.93-1.01 |
| 4^th^ quartile (ADI Range 80-100) | 0.93 | 0.90-0.97 |
|  |  |  |
